# Digestive Ferments in Intestinal Disorders of Infants

**Published:** 1889-08

**Authors:** 


DIGESTIVE FERMENTS IN INTESTINAL DISORDERS OF INFANTS.—We have examined with interest the neat and well written little work recently issued by Parke, Davis & Co. with the above title. This enterprising House set forth the fact that accurate tests show, "that they have placed upon the market a new pepsin of higher digestive power than any heretofore introduced, and possessing the exceptional advantages of being absolutely *free* from ptomaines, readily soluble, and of a digestive power hitherto unattained. Moreover, the standard of pepsin has been raised by the better manufacturers, and it is the practitioner's own fault if he is not able now to secure a preparation suited to his needs.

